# School-Based Interventions to Increase Student COVID-19 Vaccination Coverage in Public School Populations with Low Coverage — Seattle, Washington, December 2021–June 2022

**DOI:** 10.15585/mmwr.mm7211a3

**Published:** 2023-03-17

**Authors:** Tarayn Fairlie, Brian Chu, Ebony S. Thomas, Audrey K. Querns, Andie Lyons, Melissa Koziol, Janet A. Englund, Eric M. Anderson, Katherine Graff, Sara Rigel, Teal R. Bell, Sharon Saydah, Kevin Chatham-Stephens, Tara M. Vogt, Samara Hoag, Melissa Briggs-Hagen

**Affiliations:** ^1^CDC COVID-19 Emergency Response Team; ^2^Seattle Public Schools, Seattle, Washington; ^3^Public Health - Seattle & King County, Seattle, Washington; ^4^Seattle Children’s Hospital, Seattle, Washington; ^5^Washington State Department of Health.

COVID-19 can lead to severe outcomes in children (*1*). Vaccination decreases risk for COVID-19 illness, severe disease, and death (*2*). On December 13, 2020, CDC recommended COVID-19 vaccination for persons aged ≥16 years, with expansion on May 12, 2021, to children and adolescents (children) aged 12–15 years, and on November 2, 2021, to children aged 5–11 years (*3*). As of March 8, 2023, COVID-19 vaccination coverage among school-aged children remained low nationwide, with 61.7% of children aged 12–17 years and approximately one third (32.7%) of those aged 5–11 years having completed the primary series (*3*). Intention to receive COVID-19 vaccine and vaccination coverage vary by demographic characteristics, including race and ethnicity and socioeconomic status (*4*–*6*). Seattle Public Schools (SPS) implemented a program to increase COVID-19 vaccination coverage during the 2021–22 school year, focusing on children aged 5–11 years during November 2021–June 2022, with an added focus on populations with low vaccine coverage during January 2022–June 2022.^†^ The program included strategic messaging, school-located vaccination clinics, and school-led community engagement. Vaccination data from the Washington State Immunization Information System (WAIIS) were analyzed to examine disparities in COVID-19 vaccination by demographic and school characteristics and trends over time. In December 2021, 56.5% of all SPS students, 33.7% of children aged 5–11 years, and 81.3% of children aged 12–18 years had completed a COVID-19 primary vaccination series. By June 2022, overall series completion had increased to 80.3% and was 74.0% and 86.6% among children aged 5–11 years and 12–18 years, respectively. School-led vaccination programs can leverage community partnerships and relationships with families to improve COVID-19 vaccine access and coverage.

With support from local and state public health officials, SPS conducted school-located vaccination clinics at 54 schools during November 2021–June 2022. WAIIS provides monthly reports on school-required and COVID-19 vaccination coverage to SPS; these data are then linked to school system data. WAIIS data were analyzed to ascertain the monthly proportion of kindergarten through grade 12 students completing the primary COVID-19 vaccination^§^ series during December 2021–June 2022. The proportions of students completing the primary series were examined by age, race and ethnicity,^¶^ language status (monolingual versus multilingual),** use of special education services,^††^ school equity tier,^§§^ and school baseline vaccination coverage,^¶¶^ with January 2022 serving as a baseline for assessing subsequent activities to engage groups with low vaccination coverage. Qualitative and descriptive data regarding efforts by SPS to increase primary COVID-19 vaccination series completion during November 2021–June 2022 were also informally collected from approximately 10 SPS staff members and representatives of the Washington Department of Health and Public Health – Seattle & King County (PHSKC) via virtual meetings and e-mail. This activity was reviewed by CDC and was conducted consistent with applicable federal law and CDC policy.***

SPS serves approximately 50,000 students in 106 schools (Table 1). In December 2021, primary COVID-19 vaccination series completion among SPS students aged 5–18 years was 56.5% overall (Figure) and was lowest among students who were non-Hispanic Black or African American (Black) (27.9%) and multilingual (30.0%). During November–December 2021, 55 school-located vaccination clinics were held, with planning led by SPS and supported by PHSKC. These clinics included school day clinics, where children received immunizations with written parental consent but without requiring that a parent be present, and school-located regional clinics during evening or weekend hours. School-day clinics were strategically located at 41 schools selected because of size or known barriers to care.^†††^ COVID-19 vaccines were also readily available at 29 PHSKC-supported school-based health centers that provide comprehensive primary care to their students.

**TABLE 1:** Sociodemographic and school-specific characteristics of Seattle Public Schools students — Seattle, Washington, December 2021

Characteristic	No. (%)
**Total**	**50,864 (100.0)**
**Age group, yrs**	
5–11	26,341 (51.8)
12–18	24,097 (47.4)
Race and ethnicity*	
American Indian or Alaska Native, non-Hispanic	196 (0.4)
Asian, non-Hispanic	6,385 (12.6)
Black or African American, non-Hispanic	7,460 (14.7)
Pacific Islander, non-Hispanic	208 (0.4)
White, non-Hispanic	23,453 (46.1)
Hispanic or Latino	6,806 (13.4)
Multiracial, non-Hispanic	6,339 (12.5)
**Language status^†^**	
Monolingual	44,177 (86.9)
Multilingual	6,670 (13.1)
**Use of special education services^§^**	
No	43,141 (84.8)
Yes	7,706 (15.2)
**School baseline vaccination coverage^¶^**	
Low (<50%; 27 schools)	7,272 (14.3)
High (≥50%; 79 schools)	43,592 (85.7)
School equity tier**	
Tiers 1 and 2 (low equity)	17,510 (34.4)
Tiers 3 and 4 (high equity)	32,764 (64.4)

**Abbreviations**: ELL = English language learner; SPS = Seattle Public Schools.

* Race and ethnicity were based on U.S. Department of Education descriptors. Final guidance on maintaining, collecting, and reporting racial and ethnic data to the U.S. Department of Education is available at https://www.govinfo.gov/content/pkg/FR-2007-10-19/pdf/E7-20613.pdf. If the ethnicity aggregate is Hispanic or Latino, race and ethnicity is listed as Hispanic or Latino. If the ethnicity aggregate is non-Hispanic and one race category is selected, student is listed as that race. If two or more race categories are selected, student is listed as multiracial.

^†^ Language status was either multilingual, defined as student use of ELL services during the 2021–22 school year, or monolingual, with no use of ELL services in 2021–22.

^§^ Use of special education services was defined as use of an individual education plan during the 2021–22 school year.

^¶^ Defined as the proportion of a school’s students who had completed the primary COVID-19 vaccine series by the first week of January 2022. SPS school health staff members determined in January 2022 that low-coverage schools were those with a student completion rate of ≤50%, and high vaccination coverage schools were those with a completion rate of >50%.

** School equity tiers were defined as either high levels of inequity (Tier 1 or Tier 2) or low levels of inequity (Tier 3 and Tier 4), based on established SPS methodology. https://www.seattleschools.org/wp-content/uploads/2022/02/tier_methodology23.pdf

**FIGURE:**
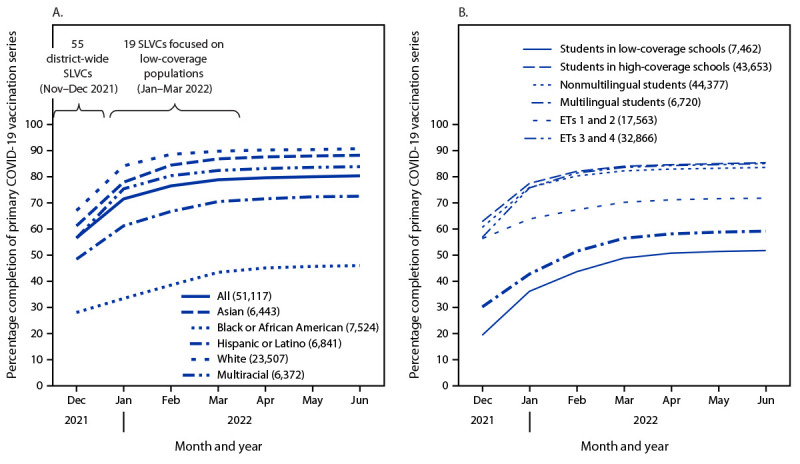
Completion of the primary COVID-19 vaccination series by students aged 5–18 years, by race and ethnicity* (A) and school coverage status,^†^ student language status, and equity tier^§^ (B) — Seattle Public Schools, December 2021–June 2022 **Abbreviations:** ET = equity tier; SLVC = school-located vaccination clinic. * Hispanic or Latino students could be of any race; other racial groups were non-Hispanic. American Indian or Alaska Native students (215), Native Hawaiian or other Pacific Islander students (197), and students with missing race and ethnicity data (18) are excluded from figure. ^†^ Low- and high-coverage schools have primary COVID-19 series completion rates of ≤50% and >50%, respectively. ^§^ Tier 1 and Tier 2 schools represent higher levels of inequity and are designated for additional support from the state; Tier 3 and Tier 4 schools have lower levels of inequity and do not qualify for additional support. https://www.seattleschools.org/wp-content/uploads/2022/02/tier_methodology23.pdf

By January 2022, primary COVID-19 vaccination series completion among SPS students aged 5–18 years had increased from 56.5% to 71.5%. After receipt of school-level student vaccination data in early January 2022, efforts during January–June 2022 focused on schools with continued low (baseline) vaccination coverage. Overall, 26 school-located vaccination clinics were conducted, including 19 during January–March 2022 that were in or near low-coverage schools (i.e., those with primary COVID-19 vaccine series completion rates of ≤50%). These clinics took place after school hours or on the weekend and were open to all SPS students and their family members. SPS administered 12,245 COVID-19 vaccine doses during November 2021–June 2022.

School-located vaccination clinics were complemented by strategies implemented to overcome cultural and linguistic barriers with families. For example, SPS conducted weekly communication with families, including email, telephone calls delivering prerecorded messages, and text messaging using TalkingPoints, a two-way communication platform that provided messaging in six languages.^§§§^ SPS also provided communications toolkits created by PHSKC in multiple languages to parent-teacher-student associations and community-based organizations to amplify messaging. Vaccine providers were selected based on their cultural competency (e.g., an independent, Black-owned pharmacy with vaccinators with facility in several African languages).

Tailored school-specific engagements were also conducted. One school used multilingual staff members from its school-based health center to administer vaccines at students’ homes or workplaces if necessary, thereby extending vaccination access beyond the school day. This school increased COVID-19 primary series completion among persons aged 11–21 years from 45% in January 2022 to 93% by June 2022. Another worked with a community health organization to organize health-related events focused on the Somali community and cohosted a school-located vaccination clinic with a local mosque. Each school used different approaches; however, all relied on school health staff members for direct family outreach.

 During the period in which SPS specifically focused on students and schools with low baseline vaccination coverage, primary COVID-19 vaccination series completion among SPS students increased 12.3%, from 71.5% in January 2022, to 80.3% by June 2022; among children aged 5–11 years and 12–18 years, coverage increased 21.3% and 3.6%, respectively (Table 2). Primary series completion increased 37.8% among Black students (from 33.3% to 45.9%), 121.8% (from 13.5% to 29.9%) among those aged 5–11 years, and 14.8% (from 53.6% to 61.5%) among those aged 12–18 years. During the same period among multilingual students, overall primary series completion increased 38.7% (from 42.6% to 59.1%), 74.6% (from 28.9% to 50.4%) and 10.6% (from 65.7% to 72.3%) among those aged 5–11 and 12–18 years, respectively. Primary series completion among students at schools with low baseline vaccination coverage also increased, from 36.0% to 51.7% (43.4% increase) overall, from 34.8% to 51.1% (46.9% increase) among students aged 5–11 years, and from 51.9% to 58.5% (11.5% increase) among those aged 12–18 years.

**TABLE 2:** Rates, absolute change, and proportional change in COVID-19 primary vaccination series completion among students aged 5–18 years, by age group, race and ethnicity, and equity tier* — Seattle Public Schools, January–June 2022

Category (average no.^†^ of students per category)	% Completion Jan 2022	% Completion Jun 2022	Absolute % change Jan–Jun 2022 (95% CI)^§^	% Change^¶^ Jan–Jun 2022
**Total (51,116)**	**71.5**	**80.3**	**8.8 (8.5–9.2)**	**12.3**
Asian, non-Hispanic (6,440)	77.9	88.2	10.3 (9.5–11.1)	13.2
Black or African American, non-Hispanic (7,524)	33.3	45.9	12.6 (11.5–13.7)	37.8
White, non-Hispanic (23,560)	84.2	90.8	6.6 (6.2–7.0)	7.8
Hispanic or Latino (6,841)	61.2	72.5	11.4 (10.3–12.4)	18.6
Multiracial, non-Hispanic (6,371)	75.3	83.9	8.5 (7.6–9.4)	11.3
Low-coverage schools (7,462)	36.0	51.7	15.6 (14.5–16.8)	43.4
High-coverage schools (43,653)	77.4	85.4	7.9 (7.6–8.3)	10.3
Monolingual (44,377)	75.9	83.6	7.7 (7.3–8.0)	10.1
Multilingual (6,720)	42.6	59.1	16.5 (15.3–17.7)	38.7
Equity tiers 1 and 2 (low equity) (17,563)	63.8	71.8	8.0 (7.3–8.6)	12.5
Equity tiers 3 and 4 (high equity) (32,866)	75.7	85.0	9.4 (8.9–9.7)	12.4
**Age group 5–11 yrs**				
**Total (25,806)**	**61.0**	**74.0**	**13.0 (12.9–13.2)**	**21.3**
Asian, non-Hispanic (2,966)	62.1	80.8	18.7 (17.2–20.1)	30.0
Black or African American, non-Hispanic (3,784)	13.5	29.9	16.4 (14.9–17.9)	121.8
White, non-Hispanic (11,995)	77.9	87.9	10.0 (9.4–10.6)	12.8
Hispanic or Latino (3,279)	45.8	62.8	17.0 (15.3–18.7)	37.1
Multiracial, non-Hispanic (3,602)	68.0	80.7	12.7 (11.4–14.0)	18.7
Low-coverage schools (6,839)	34.8	51.1	16.3 (15.1–17.5)	46.9
High-coverage schools (18,943)	70.1	82.8	12.7 (12.2–13.3)	18.2
Monolingual (21,577)	67.3	78.7	11.4 (10.9–12.0)	17.0
Multilingual (4,204)	28.9	50.4	21.6 (20.0–23.1)	74.6
Equity tiers 1 and 2 (low equity) (5,200)	33.8	48.4	14.6 (13.2–16.0)	43.2
Equity tiers 3 and 4 (high equity) (20,339)	68.0	80.6	12.6 (12.0–13.1)	18.5
**Age group 12–18 yrs**				
**Total (24,850)**	**82.7**	**86.6**	**3.9 (3.5–4.3)**	**4.7**
Asian, non-Hispanic (3,393)	91.8	94.3	2.5 (1.7–3.3)	2.7
Black or African American, non-Hispanic (3,610)	53.5	61.5	8.0 (6.4–9.6)	15.0
White, non-Hispanic (11,420)	90.9	93.6	2.6 (2.3–3.1)	2.9
Hispanic or Latino (3,459)	75.8	81.4	5.6 (4.3–6.9)	7.4
Multiracial, non-Hispanic (2,725)	85.6	88.1	2.4 (1.3–3.7)	2.9
Low-coverage schools (561)	51.9	58.5	6.6 (2.7–10.5)	12.7
High-coverage schools (24,287)	83.4	87.3	3.9 (3.5–4.3)	4.7
Monolingual (22,473)	84.5	88.1	3.7 (3.2–4.0)	4.3
Multilingual (6,720)	65.7	72.3	6.6 (4.8–8.4)	10.0
Equity tiers 1 and 2 (low equity) (17,563)	77.0	81.4	4.4 (37–5.1)	5.7
Equity tiers 3 and 4 (high equity) (12,487)	88.6	91.8	3.2 (2.7–3.7)	3.6

* Equity Tier 1 and Tier 2 schools represent higher levels of inequity and are designated for additional support from the state; Tier 3 and Tier 4 schools have lower levels of inequity and do not qualify for support. https://www.seattleschools.org/wp-content/uploads/2022/02/tier_methodology23.pdf

^†^ During January–June 2022. Categories are not mutually exclusive.

^§^ Absolute percent change from January to June 2022 for each group was compared with the hypothesized mean increase in completion of 5% for all persons aged 5–18 years, 10% for children aged 5–11 years, and 3% for persons aged 12–18 years based on COVID tracker data showing completion rate changes seen nationally during the study period using a paired t-test. The absolute change was statistically significant (p<0.05) for each group. Comparison across groups was not completed because many of the groups are not mutually exclusive.

^¶^ Percent change was calculated as follows: (proportion June 2022 − proportion January 2022) / proportion January 2022.

## Discussion

These data illustrate the potential impact of active school-based engagement on COVID-19 primary vaccination coverage among students. During the evaluation period, primary series completion in Washington among children aged 5–17 years (42.6%) was similar to national coverage in June 2022 (43.4%) (*3*,*6*). Primary series completion among children aged 5–17 years was higher in Seattle and King County (62.2%) than state-wide (42.6%); however, vaccination coverage among children aged 5–18 years in SPS (80.3%) exceeded this completion rate as well (*7*). Focused engagements during January–June 2022 to improve vaccination coverage might have contributed to high primary series completion among children attending SPS schools. Approaches included improved access via school-located vaccination clinics, outreach by school health professionals, and multimodal, multilingual communication from SPS. Coverage among all subgroups with low coverage in January 2022 significantly increased by June 2022, although overall completion remained lowest among Black and multilingual students.

Schools have the potential to play a critical role in the health of children, and can enhance access to health care services, including preventive care, particularly among those without a traditional medical home. Other studies have described the role of school-located vaccination clinics in increasing human papillomavirus and influenza vaccination coverage among students (*8*,*9*). School-located vaccination clinics can increase vaccination coverage by providing equitable access to vaccines but might be more effective when complemented with school-based messaging and other engagements to improve vaccine confidence.

The findings in this report are subject to at least six limitations. First, the intervention did not include a comparison group; thus, it is not possible to assess the relative contribution of these school-based activities to the changes in primary series completion described. Second, place of vaccination was not reported, and students might have been vaccinated at non-SPS vaccination sites. Third, monthly primary series completion data are cross-sectional, reflecting primary series completion for each subgroup at a single point in time; thus, the change in primary series completion for each subgroup cannot be attributed to individual change in behavior. Fourth, primary series completion data might be inaccurate or missing if students were vaccinated out of state. Fifth, caregivers of children in SPS might be more vaccine-confident compared with those in other U.S. populations, as suggested by high COVID-19 vaccination coverage in Seattle and King County (*6*). Finally, other interventions that affected vaccine confidence or access to care might not have been considered.

These findings illustrate and highlight the critical role that school health can play within the community. School health professionals are likely to be trusted by families (*10*). In this report, school health professionals collaborated with community and public health partners to implement strategic engagements and to facilitate opportunities for COVID-19 vaccination. School-led promotion of vaccination might improve vaccine confidence and provide support and readiness for current and future pandemics.

**Summary**

**What is already known about this topic?**

Vaccination decreases risk for COVID-19 illness, severe disease, and death. U.S. pediatric COVID-19 vaccination coverage remains low.

**What is added by this report?**

Seattle Public Schools implemented a COVID-19 vaccination program through multiple community engagements. During December 2021–June 2022, completion of the primary COVID-19 vaccination series among Seattle Public Schools students aged 5–18 years increased from 56.5% to 80.3%.

**What are the implications for public health practice?**

School health programs can provide critical information about and access to vaccinations. School health providers might also be able to leverage community partners and relationships with families to increase vaccination coverage.

## Footnotes

* These senior authors contributed equally to this report.

† CDC recommended COVID-19 vaccination for persons aged ≥16 years on December 13, 2020, for children aged 12–15 years on May 12, 2021, and for children aged 5–11 years on November 2, 2021. Booster doses for children aged 12–17 years were recommended in October 2021, and booster doses for children aged 5–11 years were recommended on May 19, 2022. During the period described, SPS focused efforts on children aged 5–11 years to maximize primary series vaccination coverage in that age group, although booster doses were available, advertised, and encouraged as well.

§ Defined as having completed the primary series of a COVID-19 vaccine approved or authorized by the Food and Drug Administration or listed for emergency use by the World Health Organization.

¶ Defined by student self-report or caregiver report at time of school registration.

** Language status as monolingual or multilingual learner; multilingual learners were defined as those using English language learning (ELL) services during the 2021–22 school year. Students using ELL services spoke a variety of other languages at home or as a first language, including Amharic, Arabic, Cantonese, Japanese, Mandarin, Oromo, Russian, Somali, Soninke, Spanish, Tigrinya, Toishanese, and Vietnamese.

†† Having an individual education plan during the 2021–22 school year.

§§ School equity tier defined using established SPS methodology. Tier 1 and Tier 2 schools represent higher levels of inequity and are designated for additional support from the state; Tier 3 and Tier 4 schools have lower levels of inequity and do not qualify for additional support. https://www.seattleschools.org/wp-content/uploads/2022/02/tier_methodology23.pdf

¶¶ The proportion of a school’s students who had completed the primary series by the first week of January 2022. SPS school health staff members defined schools with low vaccination coverage as those with a student primary COVID-19 vaccination series completion rate of ≤50%; high-coverage schools were those with a completion rate of >50%.

*** 45 C.F.R. part 46, 21 C.F.R. part 56; 42 U.S.C. Sect. 241(d); 5 U.S.C. Sect. 552a; 44 U.S.C. Sect. 3501 et seq.

††† Prioritized schools included those with >500 students, Equity Tier 1–3 schools, schools with programs for medically fragile students and with deaf and hard of hearing students, and schools whose students had less access to other large-scale vaccination opportunities.

§§§ TalkingPoints is a two-way multilingual communication platform for schools and families. SPS uses this platform to engage with families in multiple languages (Amharic, English, Mandarin, Somali, Spanish, and Vietnamese) about school-related issues, including school-located vaccination clinics for COVID-19 vaccine. https://talkingpts.org/schools
